# Assessing the mobility of Bronze Age societies in East-Central Europe. A strontium and oxygen isotope perspective on two archaeological sites

**DOI:** 10.1371/journal.pone.0282472

**Published:** 2023-03-17

**Authors:** Łukasz Pospieszny, Przemysław Makarowicz, Jamie Lewis, Anita Szczepanek, Jacek Górski, Piotr Włodarczak, Jan Romaniszyn, Ryszard Grygiel, Zdzislaw Belka

**Affiliations:** 1 Institute of Archaeology, University of Gdańsk, Gdańsk, Poland; 2 Department of Anthropology and Archaeology, University of Bristol, Bristol, United Kingdom; 3 Faculty of Archaeology, Adam Mickiewicz University in Poznań, Poznań, Poland; 4 School of Earth Sciences, University of Bristol, Bristol, United Kingdom; 5 Institute of Archaeology and Ethnology, Polish Academy of Science, Kraków, Poland; 6 Department of Anatomy, Faculty of Medicine, Jagiellonian University Medical College, Kraków, Poland; 7 Department of History and Cultural Heritage, University of Pope Jan Paweł II, Kraków, Poland; 8 Archaeological Museum in Cracow, Kraków, Poland; 9 Museum of Archaeology and Ethnography in Łódź, Łódź, Poland; 10 Isotope Research Unit, Adam Mickiewicz University, Poznań, Poland; University of California Santa Cruz, UNITED STATES

## Abstract

European Bronze Age societies are generally characterised by increased mobility and the application of isotopic methods to archaeology has allowed the rate and range of human travels to be quantified. However, little is known about the mobility of the people inhabiting East-Central Europe in the late Early and Middle Bronze Age (1950–1250 BC) whose primary subsistence strategy was herding supported by crop cultivation. This paper presents the results of strontium (^87^Sr/^86^Sr) and oxygen (*δ*^18^O) isotope analyses in the enamel of people buried in collective graves at the cemeteries in Gustorzyn and Żerniki Górne. These sites are located in Kujawy and the Nida Basin, a lowland and an upland region with clearly different environmental conditions, respectively. Both sites are classified as belonging to the Trzciniec cultural circle and were used between 16^th^ and 13^th^ centuries BC. Among the 34 examined individuals only an adult female from Gustorzyn can be assessed as non-local based on both ^87^Sr/^86^Sr and *δ*^18^O signatures in her first molar. This may indicate the practice of exogamy in the studied population but more generally corresponds with the hypothesis of limited mobility within these societies, as has previously been inferred from archaeological evidence, anthropological analysis, and stable isotope-based diet reconstruction. New and existing data evaluated in this paper show that the ^87^Sr/^86^Sr variability in the natural environment of both regions is relatively high, allowing the tracking of short-range human mobility. A series of oxygen isotope analyses (conducted for all but one individuals studied with strontium isotopes) indicates that *δ*^18^O ratios measured in phosphate are in agreement with the predicted modern oxygen isotope precipitation values, and that this method is useful in detecting travels over larger distances. The challenges of using both ^87^Sr/^86^Sr and *δ*^18^O isotopic systems in provenance studies in the glacial landscapes of temperate Europe are also discussed.

## Introduction

The Bronze Age in Europe was without a doubt a time of increased human mobility. At the core of this lies the westward migration of herders, mostly men, from the Black Sea steppes [1–3]. In addition to this, long-distance travels were undertaken for the acquisition of tin and copper, finished bronze items and other objects of prestige necessary for accelerating social and political competition [4–6]. Both warband rides and individual journeys, of both men and women, have now been revealed by various isotope analyses of human tissues preserved in the archaeological record [7–14]. It seems, however, that female exogamy remained the most common social strategy, inherited from the Neolithic [15–17]. What is more, isotopic provenance studies have also shown that some Bronze Age populations were surprisingly immobile, even when their material culture evidenced vast exchange networks [18]. Hence, variable patterns of mobility existed in Europe, and most probably they were key elements of local socio-economic strategies.

**Fig 1:**
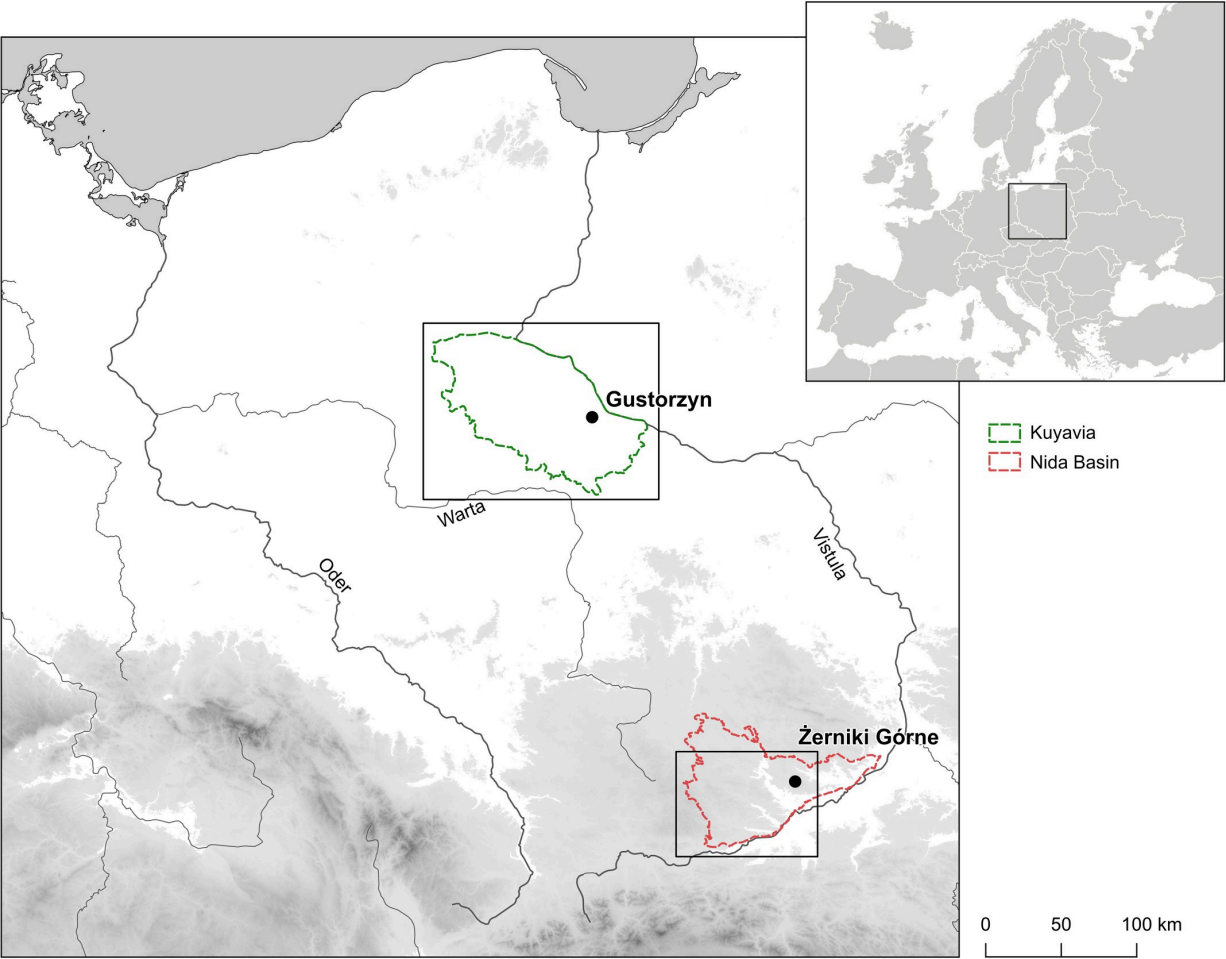
Map of East-Central Europe with location of Kuyavia (green) and Nida Basin (red), and the location of cemeteries in Gustorzyn and Żerniki Górne. Black frames correspond to the ranges of maps shown on Figs 4 and 5. Inset shows location of the study area in Europe. The map was produced with the QGIS 3.26.0 software, using elevation data downloaded from https://www.usgs.gov/.

In East-Central Europe, most mobility studies have focused on the Early Bronze Age societies, especially those representing the Únĕtice culture [17, 19, 20]. Less is known about more peripheral populations, such as those of the Trzciniec cultural circle (further referred to as TCC). In the Early and Middle Bronze Age (ca. 1950–1250 BC) the TCC societies settled what is today central and eastern Poland and western Ukraine [21, 22]. Significantly, during this period there was a clear demographic increase, which resulted in the appearance of thousands of settlements, including some which were stable and long-lasting, and more than 100 accompanying cemeteries. The lifestyle of TCC societies was much more settled and stable, compared to preceding groups from the Neolithic-Bronze Age transition period in this area [23–28]. In the north it was the region of Kuyavia (Fig 1) where a high concentration of TCC sites has now been found, pointing towards the presence of a relatively large population, exploiting mainly the fertile black earth (*chernozems*) developed in the moraine plains. From there, they most probably migrated south-south-east, along the Warta River [22], and the second-largest cluster of sites is located in the southern and south-western Nida Basin, part of the Lesser Poland Upland (Fig 1). Here they mainly settled brown and black soils formed on loess uplands.

Most importantly, in both regions, stock-rearing was the primary economic practice, supported by crop cultivation. The deceased were often equipped with grave goods resembling the trans-Carpathian style, from the Otomani-Füzesabony circle, and later from the Piliny culture [29, 30]. Such similarities in material culture have been interpreted not as a result of migration or exogamy on a mass scale, but rather as permanent interregional contacts, leading to the exchange of goods and cultural patterns, transfer of knowledge and know-how, and translocation of small groups of people only. Such connections cannot be traced by ancient DNA studies, as biological differences between these groups were too small [31]. Here, isotopic provenancing can offer an insight into human mobility at an unprecedented spatial resolution.

Currently, strontium (Sr) and oxygen (O) isotope analyses are the most robust and effective tools used in archaeology for mobility and geographic provenance studies [7, 8, 15, 17, 32–34]. In this article, we present the results of anthropological and isotopic analyses of 35 individuals buried in communal graves at two TCC cemeteries, located in distant regions of Poland–Gustorzyn in Kuyavia and Żerniki Górne in the Nida Basin (Fig 1). These regions were distinct in terms of environmental conditions and trajectories of socio-cultural development, shaping people’s lifeways and subsistence strategies [23, 24, 26]. The number (*n* = 32) of people buried in the preserved part of the cemetery in Gustorzyn is small when compared to other Bronze Age sites in Europe. However, this is the largest TCC cemetery in Kuyavia and elsewhere in the lowlands of northern Poland. The cemetery in Żerniki Górne is not only the largest among TCC cemeteries in the Nida Basin but also one of the largest Bronze Age cemeteries in south-eastern Poland. Hence, this study covers two of the most important and representative “Trzciniec” sites ever excavated.

The application of Sr and O isotope analyses for a relatively large series of samples from two representative sites is an opportunity to gain insights into mobility patterns of communities following similar subsistence strategies but living in different environments: lowlands and uplands. Furthermore, the parallel application of two isotope systems for virtually all individuals, with the use of the same enamel samples, allows the assessment of the effectiveness of these systems in identifying non-local individuals in the given geological and geographic settings. In the area of today’s Poland O isotope systems have mainly been used for studying medieval and post-medieval populations [14, 35–38]. For prehistoric populations its use has been much more limited [39–42]. Moreover, some of the results have been problematic and are difficult to interpret [40]. Hence, the aims of this study are: (i) to estimate the local Sr and O isotope ranges for Kuyavia and the Nida Basin, two areas with clearly different geologies and environmental conditions, (ii) to unravel the patterns of mobility within two contemporary Bronze Age populations from these two areas, and in turn (iii) to evaluate the efficiency of O isotope analyses in searching for non-local individuals from two of the most archaeologically important regions of prehistoric East-Central Europe. To aid in (iii), a compilation of published oxygen isotope ratios in tooth enamel in the study area is presented with all isotope ratios normalised to the same isotopic reference scale (VSMOW).

### Trzciniec cultural circle. An overview

Studies based on *site catchment* analyses have allowed the settlement model of TCC communities in Kuyavia to be reconstructed [26]. It was organized around so-called micro-regions comprising central settlements, smaller settlements (campsites) and a neighbouring cemetery. Most of the human activity took place within a radius of ca. 2 km from the central settlements. Most plausibly, after their emergence in the northern lowlands ca. 1800 BC, “Trzciniec” people migrated south-east, colonised the uplands of today’s south-eastern Poland and western Ukraine, keeping those settlement patterns developed in the north. The mainstays of their subsistence strategy, both in lowlands and uplands, was animal husbandry and plant cultivation. This is confirmed by grazing markers visible in the pollen records of various landscape zones: on wet floodplain meadows of river terraces, on dry meadows in the deforested parts of areas of high relief, on the edges of forests and in forest clearings. Ruminants such as cattle and sheep/goat dominated the structure of the herds while pigs and other domesticates were less important [22, 26]. The presence of cereal pollen in palynological profiles indicates a complementary role of crops, mainly wheat and barley [22, 43]. Direct radiocarbon dating of charred broomcorn millet grains and stable isotope analyses of human remains revealed that since the middle of 15^th^ c. BC this plant was cultivated and consumed in the uplands while in the lowlands it was most probably unknown before the 13^th^ c. BC [44, 45].

**Fig 2:**
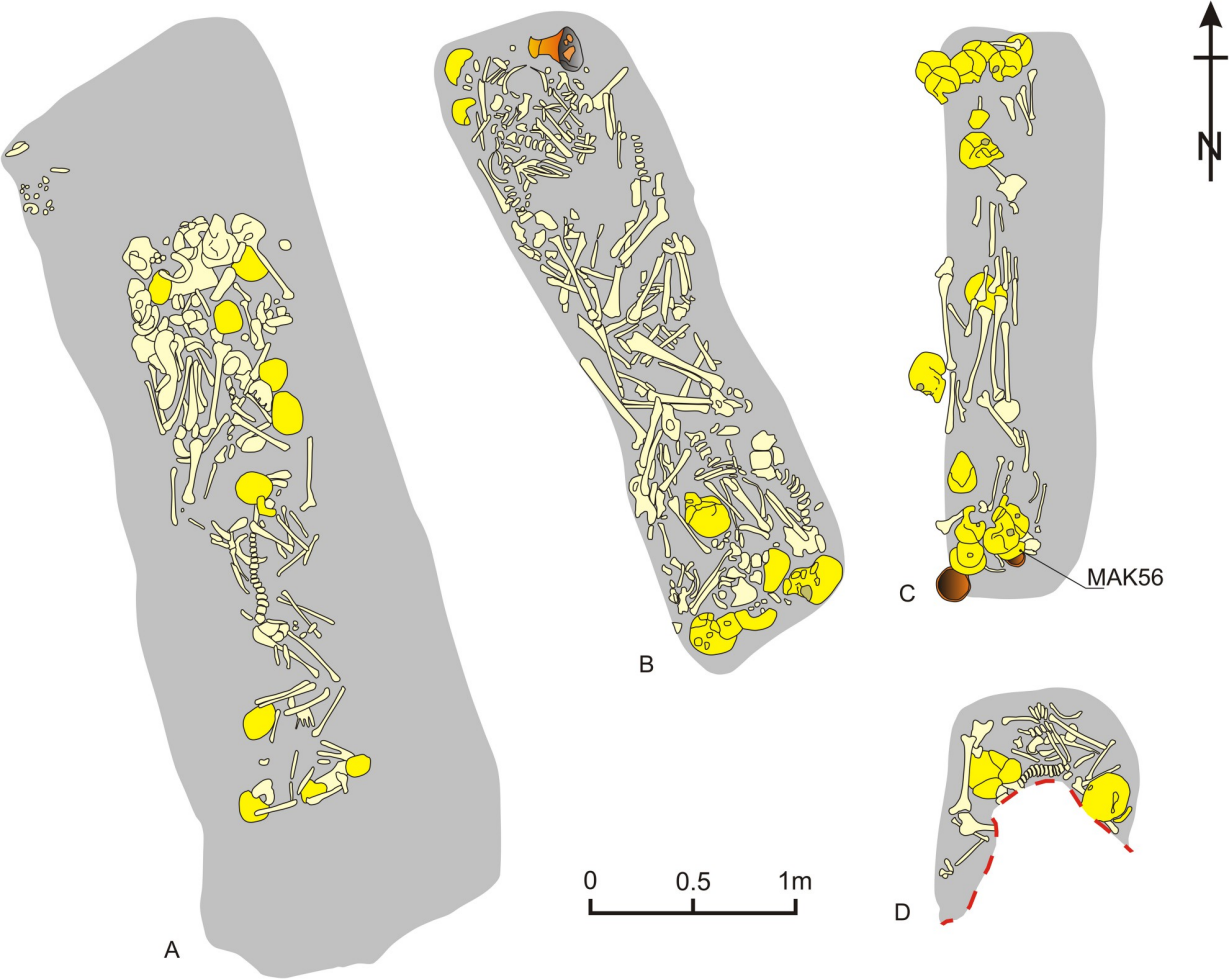
Simplified outlines of graves nos. 69 (A) and 99 (B) in Żerniki Górne (after [51]) and nos. 4 (C) and 5 (D) in Gustorzyn (after [48], simplified). Human and animal bones are marked in yellow. In Gustorzyn darker shade marks bones described by excavators as belonging to human skulls, in Żerniki Górne human skulls were solely recognised basing on published figures; stone constructions were omitted.

The characteristic form of burial rite among TCC societies was collective graves [22, 46–48]. The deceased were placed in antithetic (antipodal) order, with heads on the shorter side of a pit and legs pointing to its centre (Fig 2). Human bones in such graves are usually found arranged in piles, and only some skeletons or parts of skeletons were kept in anatomical order. How such arrangements were built is difficult to reconstruct because of either lack of accurate field documentation or the inability to distinguish between human agency and post-depositional processes. It seems, however, that bodies were placed in the graves shortly after death, i.e., with preserved soft tissues and joint connections. This was preceded by moving the remains of previously buried individuals, likely at various stages of decomposition, to make space for the new interments. Radiocarbon dating of tombs from numerous cemeteries in Poland and western Ukraine indicates that the collective graves were in use from periods of 10 years to a maximum of 200–250 years [47, 49, 50]. They could therefore be used by several to even a dozen generations or more.

## Materials

### Gustorzyn

The cemetery at Gustorzyn site 1 is located within the Kuyavia Plain, known also as the Kuyavia Moraine Plateau. It lies at the border of the villages of Gustorzyn and Polówka, on a promontory of a flat moraine plateau, separated from the south by the Zgłowiączka river valley and from the west and north by a small valley with a tributary to the Zgłowiączka. Two communal graves (nos. 4 and 5; Fig 2) were discovered during rescue excavations in 1981 on the north-western outskirts of an active gravel pit [52]. In total, at least 32 people were buried here but it is obvious that the original number of individuals was higher. The general characteristic of the two preserved graves is presented in S1 Text.

Eight of the 17 individuals included in this study were ^14^C-dated by Accelerator Mass Spectrometry (AMS) and the remaining collagen was submitted for carbon (*δ*^13^C) and nitrogen (*δ*^15^N) stable isotope analyses. The results have been published elsewhere [45, 53] and summarised in Table 1. They indicate that both graves were in use between approx. middle 15^th^ and early 13^th^ centuries BC, and that the diet of dated individuals was based mainly on C_3_ plants and terrestrial animals. For both radiocarbon dating and stable isotope analyses, outer layers of petrosal bones were sampled. Inner parts would normally reflect diet in the early years of life [53]. However, the outer parts most probably inform about later stages of infancy, most certainly after weaning [54], as evidenced by relatively low *δ*^15^N values (≤ 10.8 ‰) (Table 1).

**Table 1:** Detailed context information, strontium (^87^Sr/^86^Sr) and oxygen (*δ*^18^O_P-VSMOW_) isotope data, AMS radiocarbon ages and bone bulk collagen carbon (*δ*^13^C) and nitrogen (*δ*^15^N) isotopic data for humans from cemeteries in Gustorzyn and Żerniki Górne. Anthropological data for Gustorzyn after [48, 56]. All ^14^C dates were calibrated with OxCal 4.4 [123] using the IntCal 2020 curve [124].

Site	Feature/individual	Age (years)	Sex	Tooth	Sample ID	^87^Sr/^86^Sr	Error ±	*δ*^18^O_P-VSMOW_	1 SD	^14^C lab ID	BP	SD	*δ*^13^C_coll_	*δ*^15^N_coll_	95.4% (2σ)
															**cal. BC**
Gustorzyn	grave 4/skull 1	12–14	?	UM1	MAK67	0.712010	0.000016	18.3	0.10						
Gustorzyn	grave 4/skull 3	20-x	m	LM1	MAK52	0.711650	0.000010	17.2	0.11	Poz-101065	3030	40	–19.9	10.1	1407–1131
Gustorzyn	grave 4/skull 4	30–40	m	UP1	MAK74	0.711760	0.000009	17	0.08	Poz-142716	3070	35			1421–1226
Gustorzyn	grave 4/skull 5	18–20	?	LM1	MAK66	0.711767	0.000010	17.7	0.10	Poz-142717	3075	35			1423–1230
Gustorzyn	grave 4/skull 6	25–35	?	M1	MAK55	0.711245	0.000009			Poz-142718	3065	35			1416–1226
Gustorzyn	grave 4/7	5–6	?	LM1	MAK57	0.711262	0.000008	16.9	0.11						
Gustorzyn	grave 5/1	20–30	m	UM1	MAK62	0.711539	0.000011	17.2	0.04						
Gustorzyn	grave 5/2	20–30	m	UM1	MAK54	0.712276	0.000010	17.4	0.10						
Gustorzyn	grave 5/3	40–50	m	UP1	MAK53	0.711718	0.000011	18.3	0.19						
Gustorzyn	grave 5/5	5–6	?	M1	MAK64	0.711670	0.000009	16	0.08						
Gustorzyn	grave 5/6	20–30	f	LM1	MAK56	0.709822	0.000010	15.9	0.09	Poz-142719	3045	35			1412–1213
Gustorzyn	grave 5/7	20–30	f	LM1	MAK63	0.711595	0.000013	17.8	0.22	Poz-101465	3160	35	–20.3	9.7	1506–1310
Gustorzyn	grave 5/10	30–40	?	LM1	MAK60	0.712488	0.000010	18.3	0.10						
Gustorzyn	grave 5/11	20–30	f	UM1	MAK59	0.711865	0.000013	17.6	0.04	Poz-101466	3185	35	–20.3	10.8	1528–1401
Gustorzyn	grave 5/13	20–30	f	UM1	MAK65	0.711452	0.000010	17.1	0.04						
Gustorzyn	grave 5/14	20–30	m	M1	MAK61	0.711954	0.000025			Poz-101025	3045	35	–20	9.8	1409–1214
Gustorzyn	grave 5/15	10–12	?	UM1	MAK58	0.711170	0.000010	17.8	0.15						
Żerniki Górne	69/141	16–18	f	LM1	MAK78	0.708406	0.000010	16.3	0.13						
Żerniki Górne	69/142	20–30	f	LM1	MAK80	0.708529	0.000012	15.7	0.13	Poz-107545	3255	35	–19.9	10.9	1616–1448
Żerniki Górne	69/skull13, n151	20–30	f	UM1	MAK81	0.708159	0.000010	16.2	0.06						
Żerniki Górne	69/146	18–20	f	LM1	MAK82	0.708948	0.000010	15.7	0.05						
Żerniki Górne	69/skull 6	40–50	m	UM1	MAK83	0.708248	0.000010	16.5	0.05	Poz-107541	3255	35	–19.9	9.4	1616–1448
Żerniki Górne	69/157	20–30	m	UM1	MAK84	0.708631	0.000016	16	0.10						
Żerniki Górne	99/68/II	40–55	m	LM1	MAK87	0.708736	0.000011	16.3	0.10	Poz-92375	3055	30	–20.1	10	1407–1231
Żerniki Górne	99/68/I	40–50	m	UM1	MAK88	0.708779	0.000013	16.1	0.17						
Żerniki Górne	99/skull IV	10–12	?	UM1	MAK89	0.708557	0.000016	16.4	0.16						
Żerniki Górne	62/N75	6–7	?	UM1	MAK90	0.708786	0.000010	16.2	0.12	Poz-101468	3170	35	–20.1	10.2	1511–1321
Żerniki Górne	62/535	20–30	f	UM1	MAK91	0.709006	0.000014	15.8	0.08	Poz-104933	3230	30	–20.1	9.8	1608–1432
Żerniki Górne	62/6	20-x	?	UM1	MAK92	0.708706	0.000010	15.9	0.12	Poz-93454	3235	35	–19.9	9.8	1611–1434
Żerniki Górne	12/A	20–30	m	LM1	MAK93	0.708244	0.000012	16.5	0.18						
Żerniki Górne	12/C	40–50	m	UM1	MAK94	0.708711	0.000011	15.3	0.01						
Żerniki Górne	12/5e	40–50	m	UM1	MAK95	0.708352	0.000011	15.9	0.16						
Żerniki Górne	12/4d	20-x	m	LM1	MAK96	0.708401	0.000011	16.1	0.17	Poz-92373	3140	30	–20.2	9.8	1497–1305
Żerniki Górne	10	20-x	f	LM1	MAK97	0.709136	0.000018	16	0.05	Poz-107538	3275	35	–20.3	9.4	1631–1455
Żerniki Górne	10/skull 9	35–40	f	M1	MAK98	0.708538	0.000009	16.2	0.10						

### Żerniki Górne

The cemetery in Żerniki Górne site 1 is located in the central Nida Basin, which is a part of the Lesser Poland Upland. The site is located on a promontory of a loess plateau, falling south towards a small valley, drained by a little watercourse and surrounded by ridges of limestone formations (Fig 5). It was excavated from 1965 to 1968 [51]. The TCC necropolis was established on the location of older cemeteries, used firstly by Final Neolithic Corded Ware culture communities and then by Early Bronze Age Mierzanowice culture groups [51, 55]. At the top and partly on the slopes of the hill, four stone circles (mainly blocks of marl deposits) with a diameter of ca. 4.0 to 6.0 m have been laid. Near them, five pits filled with burnt animal bones and fragments of ceramic vessels were unearthed. The stone circles and pits were covered by a mound (the assumed central burial was destroyed by a modern pit), with the oldest graves located nearby and younger ones dug into it [47]. In total, 16 graves were discovered in which at least 170 individuals were buried. The five graves chosen for this study are described in more detail in S1 and graves nos. 69 and 99 are shown on Fig 2. The Bayesian modelling of 25 ^14^C AMS dates indicates that the cemetery was used for 140 to 310 years, i.e., for a maximum of eight to ten generations [45].

Between one to three individuals from each grave were AMS ^14^C-dated and targeted with *δ*^13^C and *δ*^15^N analyses (Table 1) [45, 47]. In all but one case, the outer parts of petrosal bones were sampled. The radiocarbon-dated humans lived between the middle 16^th^ and late 15^th^ centuries BC, except one from the end of the 14^th^ century BC. As in Gustorzyn, their main source of dietary protein was C_3_ plants and animals with no evidence of admixture of millet and no signal of breastfeeding was detected among the children studied [45]. However, it should be noted that ten more individuals from Żerniki Górne were AMS ^14^C-dated and one of them (individual 1 from grave no. 54), who died in the second half of the 14^th^ century BC, was a “millet-eater” [45].

## Methods

### Anthropological analyses

Human remains from graves nos. 4 and 5 in Gustorzyn were first anthropologically analysed in the early 2000’s [56] while finds from Żerniki Górne were investigated by A. and A. Wierciński and published in an archaeological monography of the site [51]. These analyses were later verified during material sampling for this study. The anthropological examination was prepared according to the classical morphological methods with the special modification for collective graves. The first stage was a selection of bones belonging to one individual. As a result of the similar age at death for some individuals, it was not possible to assign all remains. After the separation of individual skeletons, the anthropological determination was conducted as for single burials. In the case of child skeletons, the analysis also included the degree of ossification and the size of long bones [57, 58], as well as the development of dentition [59]. The age of adult individuals was established on the basis of the fusing of cranial sutures and dental attrition. Sex was determined only for adult individuals and was based on a comprehensive analysis of dimorphic features on the skull and pelvis [60].

### Strontium isotope analyses

The principles of using Sr isotopes as a tool for provenancing archaeological individuals have been described elsewhere [61–64] and thus only a summary is presented here. In provenance studies, the ^87^Sr/^86^Sr ratio is of primary interest as it does not change (fractionate) in any measurable way when Sr is transferred from the local source rocks through the natural environment and biosphere [65, 66]. However, because various components of the environment usually differ from each other in their ^87^Sr/^86^Sr ratios [67, 68], the Sr isotope composition of organic tissues (plants, animals, and humans) results from the mixing of strontium derived from local Sr reservoirs, i.e., the geological substrate and surface water (and/or groundwater). The Sr isotope composition of human enamel, for example, represents a weighted average of Sr derived from all the food and water ingested while the enamel was mineralized [69, 70], i.e., in the case of molars, in childhood. Thus, human enamel reaches an ^87^Sr/^86^Sr ratio, which is between the composition of the geological substrate and the drinking water [61, 64, 71] and is usually very close to that of the water.

To identify local vs. non-local individuals in a studied human population it is necessary to establish the range of “local” Sr isotope ratios available to plants, animals and humans within a given ecosystem. This can be done by analysing the Sr isotope composition of the geological substrate (rocks, sediments, soils), surface and/or ground waters, and samples of plants and animals. Recent studies, however, showed that the Sr isotope system of modern environments, i.e., its surface waters, flora and fauna, can be influenced by inputs of Sr from various anthropogenic sources [67, 72–74]. Therefore, caution is needed if Sr isotope signatures of modern samples are used. Alternatively, subfossil samples of animals (“archaeofauna”) can be analysed. This approach is usually hampered by the availability of animal remains from settlements located close to cemeteries. Moreover, domestic archaeological animals may have been imported to the studied sites and therefore a non-local provenance of “archaeofauna” can never be excluded beforehand [61, 71, 75].

In the case of the two sites covered by this study samples of archaeological fauna were not available to us. Hence, we estimated the local Sr isotope background by (i) reviewing the geological substrates of both cemeteries and their surrounding areas, (ii) reviewing published data on Sr isotope composition of sediments and waters in both regions, (iii) reviewing published data on Sr isotope composition of archaeological humans and animals from other sites in both regions.

Strontium isotope analyses were carried out in the Isotope Laboratory at the Adam Mickiewicz University in Poznań, Poland. The mechanically isolated enamel was first cleaned in an ultrasonic bath in ultrapure water to remove sediment particles. Afterwards, about 10 mg of powdered enamel was treated sequentially with 2 ml of 0.1 N ultrapure acetic acid (5 times) to eliminate the diagenetic Sr contamination, according to the procedure described by [76]. Subsequently, the samples were dissolved on a hot plate (~100°C, overnight) in closed PFA vials using 1 N HNO_3_. The miniaturized chromatographic technique developed by [77] and modified by [78] was applied for Sr separation. Purified Sr was loaded with a TaCl_5_ activator on a single Re filament and analyzed in dynamic multi-collection mode on a Finnigan MAT 261 mass spectrometer. Samples were corrected for mass-dependent fractionation by internal normalisation to a fixed ^86^Sr/^88^Sr of 0.1194 using an exponential mass bias law [79, 80]. During this study, the NIST SRM 987 Sr standard yielded ^87^Sr/^86^Sr = 0.710224 ± 11 (2σ, *n* = 15). The measured ratios were normalized to a nominal value of 0.710240 for the standard NIST SRM 987. Total procedure blanks were less than 70 pg.

### Oxygen isotope analyses

The principles of using O isotopes in archaeological provenance studies have been detailed elsewhere [81, 82] and only a summary is given here. The O isotope composition (expressed as *δ*^18^O) in animal and human tissues is related to the isotopic composition of ingested water, primarily controlled by the local meteoric water composition, with minor contributions from food, usually at the level of 30% [83–88].

*δ*^18^O in local meteoric water are products of fractionation in the hydrological cycle and are dependent mainly on local temperature and, to a lesser degree, on local altitude, latitude, humidity and distance from the sea [89–97]. Hence, if the measured *δ*^18^O values of human tissues are different than the *δ*^18^O values of local meteoric water, the non-local origins of this human can be assessed. However, as *δ*^18^O values in precipitation are correlated with temperature they can vary seasonally within the same geographic area [91]. Moreover, they can be affected by long-term climate changes. As a consequence, caution is needed when comparing oxygen isotope ratios between humans or animals living in the same location but in different geological and archaeological periods. Consequently, *δ*^18^O values measured for mineralized human and animal tissues can serve for reconstructing past climatic conditions (palaeotemperatures) [98–101].

A common approach is to compare *δ*^18^O values measured for archaeological animals and humans to modern *δ*^18^O precipitation values, as *δ*^18^O precipitation values reconstructed for past climates are rare. It is, however, advised to use such values only as approximation as they may not reflect the past values accurately [81].

In provenance studies using dental enamel and bone one measures the relative abundance of ^16^O and ^18^O in the sample (^18^O/^16^O) and this is typically expressed in permil notation (‰) relative to standard composition using the following formula:

$$\delta^{18}O(‰)=({[}^{18}O{/}^{16}O_{sample}{/}^{18}O{/}^{16}O_{standard}]‐1)\times 1000$$ (1)

The bioapatite lattice in bones and tooth enamel of humans and other mammals contains two main locations where oxygen atoms are found. These are the oxygen bound to the phosphate group (PO_4_^3–^) and that bound to the structural carbonate (CO_3_^2–^), with an additional minor component of hydroxyl (OH^–^) oxygen. Both the phosphate and carbonated bound oxygen can be isolated and used for isotope analysis and are commonly reported as *δ*^18^O_P_ and *δ*^18^O_C_, respectively [99, 102–105]. *δ*^18^O_P_ is sourced from body water, *δ*^18^O_C_ comes from blood biocarbonate (*δ*^18^O_BC_) which is isotopically equilibrated with body water and depends on the temperature at which it is precipitated [86, 87, 95, 106–108].

The human body isotopically fractionates oxygen between ingested water and body water [83, 86, 109]. However, the fractionation appears to be linear, and there is a consistent offset of ~18 ‰ between the *δ*^18^O of body water and PO_4_ in bioapatite, and a further offset of ~8 ‰ between PO_4_ and CO_3_ bound oxygen [99, 106]. Nevertheless, deviations have been reported and they have been attributed to, for instance, differences in food sources or water sources and volume [83, 85, 86, 95, 108, 110] or processing the drinking water by stewing, boiling, fermentation or distillation [111]. Most importantly, isotopic fractionation between the mother’s body and breast milk leads to elevated O isotope values in tissues forming during childhood, and the reported ^18^O enrichment ranges from 0.5 to ~2 ‰ [112, 113].

Oxygen isotope nomenclature can be further complicated by the two different isotopic scales that have been used for carbonates and waters. Historically, *δ*^18^O values were first expressed relative to PDB (Pee Dee Belemnite) carbonate standard which is now exhausted. Today two other standards are used, VPDB (Vienna Pee Dee Belemnite) and more common VSMOW (Vienna Standard Mean Ocean Water). The relationship between the two isotope scales is well understood and most authors use Coplen’s [114] conversion equation:

$$VSMOW=1.03091\times \delta^{18}O\;VPDB+30.91$$ (2)

The relationship between *δ*^18^O_P_ values in human bioapatite and *δ*^18^O values in drinking water (referred to as *δ*^18^O_DW_) is well established [87, 96, 115]. Daux and collaborators [109] provide a series of equations for calculating drinking water oxygen isotope values from measured *δ*^18^O_P_ values using either (i) tap water values or (ii) OIPC (Online Isotopes in Precipitation Calculator) [115] precipitation values. Combining this data with previously published work [86, 95, 116] provides a single equation for converting between *δ*^18^O_P_ and *δ*^18^O_DW:_

$$\delta^{18}O_{DW}=1.54(\pm 0.09)\times \delta^{18}O_{P}-33.72(\pm 1.51)$$ (3)

Pollard and collaborators in their meta-study [117] provide another equation termed “Classical regression” which gives a regression with a very similar slope and intercept:

$$\delta^{18}O_{DW}=1.55\times \delta^{18}O_{P}-33.49$$ (4)

A direct relationship between *δ*^18^O_P_ and *δ*^18^O_C_ from human tooth enamel has been established by a study of Chenery and collaborators [81]. They provide a conversion equation from *δ*^18^O_C_ to *δ*^18^O_P_:

$$\delta^{18}O_{P}(VSMOW)=1.0322\times \delta^{18}O_{C}(VSMOW)–9.6849$$ (5)

Combining (3) and (5) allows for the direct conversion from enamel *δ*^18^O_C_ to *δ*^18^O_DW_:

$$\delta^{18}O_{DW}=1.590\times \delta^{18}O_{C}(VSMOW)–48.634$$ (6)

In this paper, all oxygen isotope data are reported on the VSMOW scale. For literature data, oxygen isotope data reported on the VPDB scale are first converted to VSMOW scale using (2) and *δ*^18^O_C_ values are converted to *δ*^18^O_P_ values using (5). Following this, *δ*^18^O_P_ values are converted to *δ*^18^O_DW_ values using (3).

However, whilst the conversion equations between bioapatite *δ*^18^O values and drinking water *δ*^18^O values described above can be easily implemented, the full treatment of the data requires that the uncertainty (error) in the regression lines be propagated into the final uncertainty on the *δ*^18^O_DW_. When this is considered, it is clear that final calculated *δ*^18^O_DW_ values have a relatively large error, above 1 ‰, in some cases reaching ± 3.5 ‰ [109, 117]. Such errors are substantially higher than the analytical precision that can be obtained on replicated *δ*^18^O of bioapatite samples (0.03 to 0.2 ‰ 1σ) and in some cases may well be higher than *δ*^18^O variability within local human populations or even mask outstanding values received for non-local individuals. Hence, as suggested in the literature [117–119] to study human mobility within and between populations only *δ*^18^O values measured directly in bioapatite are compared and predicted *δ*^18^O values in precipitation are used only as a background reference.

In this study *δ*^18^O_P_ analyses were conducted in the GeoZentrum Nordbayern at the Friedrich-Alexander University in Erlangen-Nürnberg, Germany. Powdered enamel remaining after analyses in Poznań was first transferred to Erlangen. Chemical conversion of apatite bound phosphate into trisilverphosphate (Ag_3_PO_4_) was performed according to the method described by Joachimski and collaborators [120]. *δ*^18^O analyses were performed using a TC-EA (high-temperature conversion-elemental analyzer) coupled online to a ThermoFinnigan Delta V Plus mass spectrometer. 0.2 to 0.3 mg Ag_3_PO_4_ was weighed into silver foil and transferred to the sample carousel of the TC-EA. At 1450°C, the silver phosphate was reduced, and CO formed as the analyte gas [121]. CO was transferred in a helium stream through a gas chromatograph via a Conflo III interface to the mass spectrometer. Samples as well as standards were generally measured in triplicate. The measurements were calibrated by performing a two-point calibration [122] using NBS 120c (21.7 ‰) and a commercial Ag_3_PO_4_ (9.9 ‰). A laboratory standard was used as a control standard and processed together with the samples. All standards were calibrated to TU1 (21.11 ‰) and TU2 (5.45 ‰) [121]. External reproducibility, monitored by replicate analyses of samples as well as the laboratory standard was ± 0.11 ‰ (1σ; *n* = 3). The average oxygen isotope composition of the internationally distributed standard NBS 120c was measured as 21.70 ± 0.06 ‰ (1σ; *n* = 8).

## Results

Age at death was determined for all 17 individuals from the two graves in Gustorzyn and for 10 of them also sex was identified (Table 1). In the case of Żerniki Górne age was also assessed for all humans and sex was not determined only in the case of the two youngest individuals (Table 1).

^87^Sr/^86^Sr and *δ*^18^O_P-VSMOW_ isotope data were received for all analysed individuals; the results are presented in detail in Table 1 and summarised in Table 2. Table 1 brings also detailed information on each sample and its archaeological context, as well as AMS radiocarbon ages and bone bulk collagen carbon (*δ*^13^C) and nitrogen (*δ*^15^N) isotopic values for 12 individuals.

**Table 2:** Descriptive statistics of isotopic data for humans from Gustorzyn and Żerniki Górne.

	^87^Sr/^86^Sr						*δ*^18^O_P-VSMOW_					
Site	*n*	mean	median	sd	min	max	*n*	mean	median	sd	min	max
Gustorzyn	17	0.71160	0.71167	0.00058	0.70982	0.71249	15	17.37	17.40	0.74	15.90	18.30
Żerniki Górne	18	0.70860	0.70859	0.00027	0.70816	0.70914	18	16.06	16.10	0.31	15.30	16.50

### Gustorzyn

**Fig 3:**
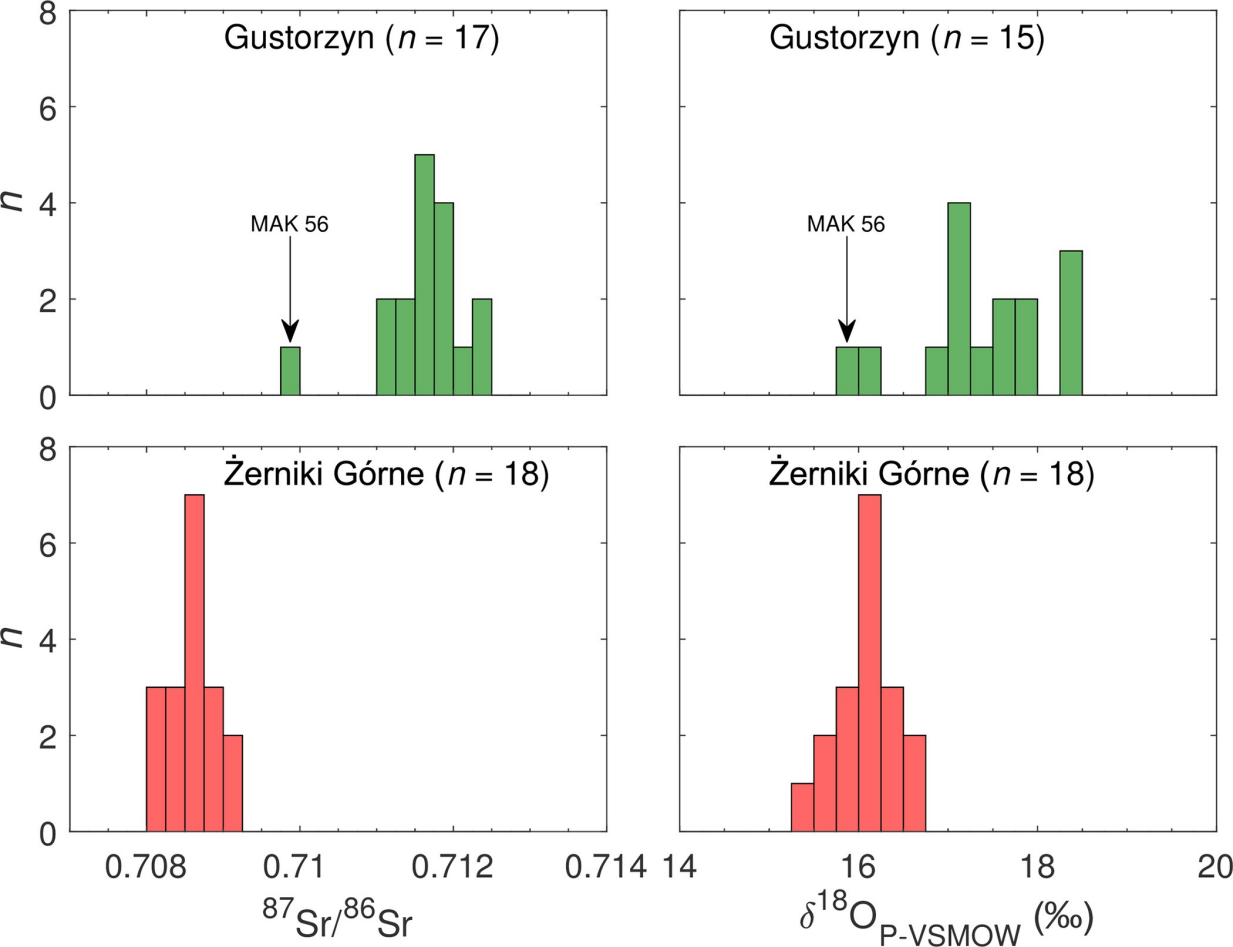
Strontium (^87^Sr/^86^Sr) and oxygen (*δ*^18^O_P-VSMOW_) isotope composition in tooth enamel of humans from cemeteries in Gustorzyn and Żerniki Górne.

The ^87^Sr/^86^Sr values of the human enamel vary within a relatively wide range from 0.7098 to 0.7125 (Tables 1 and 2; Fig 3). However, there is a single outlier in the data set–a female aged 20–30 from grave 5 (MAK56), who exhibits the most unradiogenic Sr isotope signature. Discounting this value (0.7098), the population at Gustorzyn shows in fact a narrow range, from 0.7112 to 0.7125, and there are no significant differences in the Sr isotope composition of males (0.7112–0.7123, *n* = 7) and females (0.7115–0.7119, *n* = 3). Due to the small number of samples, it is difficult to say whether this feature was also characteristic of the entire population of Gustorzyn. The range of values measured for juveniles of undetermined gender (0.7113–0.7125) only slightly exceeds the range of males.

*δ*^18^O_P_ ratios were measured for 15 out of 17 samples subjected to ^87^Sr/^86^Sr analyses. The *δ*^18^O_P_ results range from 15.9 ‰ to 18.3 ‰ and have a normal distribution (Shapiro-Wilk test, W = 0.900, Two-tailed p-value = 0.115, α = 0.050). However, two individuals have slightly lower *δ*^18^O_P_ ratios. First (15.9 ‰) was obtained for the above-mentioned female from grave no. 5 and the second (16.0 ‰) was measured for the child (individual 5, aged 5–6) from the same feature. In the case of other individuals, there are no clear differences in *δ*^18^O_P_ values related to age and/or sex. In case of juveniles, *δ*^18^O_P_ values range from 16.9 ‰ to 18.3 ‰ (*n* = 3), for males it is a range from 17.0 ‰ to 18.3 ‰ (*n* = 7), and for females between 17.1 ‰ and 17.8 ‰ (*n* = 3).

### Żerniki Górne

^87^Sr/^86^Sr and *δ*^18^O_P_ isotope signatures were measured for enamel samples taken from all 18 individuals. The ^87^Sr/^86^Sr values vary within a very narrow range from 0.7082 to 0.7091 and there is no correlation between the values obtained for the examined individuals and their age and/or sex (Table 2; Fig 3). ^87^Sr/^86^Sr ranges for females and males are almost identical, from 0.7082 to 0.7091 (*n* = 8), and from 0.7082 and 0.7088 (*n* = 8), respectively.

*δ*^18^O_P_ values range from 15.3 ‰ to 16.5 ‰ and have a normal distribution (Shapiro-Wilk test, W = 0.949, Two-tailed p-value = 0.449, alpha = 0.050). In case of females, the range is 15.7–16.3 ‰ (*n* = 8), for males 15.3–16.5 ‰ (*n* = 8), and for children 16.2–16.4 ‰ (*n* = 2). Hence, there is no correlation between the measured *δ*^18^O_P_ values and the sex and/or age of studied individuals.

## Discussion

To discern patterns of mobility in humans based on their isotopic signatures it is necessary to assess to local ranges of both Sr and O isotopes within the area of their possible residency. Using the archaeological model for TCC sites it can be assumed that such areas spread ca. 2 km around the central settlement and the nearby cemetery, covering an area of ca. 12 km^2^. However, the effectiveness of these isotopic systems in detecting non-local individuals will depend on the variation in the Sr isotope composition of the local environment and O isotopes in precipitation over a wider area, outside that of the central site catchment. It also can be assumed that more distant journeys took place between the nearest clusters of contemporary sites, usually termed microregions.

In the case of Gustorzyn, such clusters are mainly located within the region of Kuyavia. Hence, to infer whether travels within that region would be isotopically detectable an overview of its geological setting is discussed below. The case of Żerniki Górne is more challenging, despite it being the largest TCC cemetery in the Nida Basin its surroundings have not been systematically surveyed. More data is available for the western part of the Nida Basin, however, despite the lack of known settlements in its eastern part, it can be assumed that the entire region was equally exploited in the Bronze Age and its geological characteristic is described below as well.

The geological reviews of both regions are supplemented by a review of published Sr isotope data for sediments and waters, archaeological humans and fauna from other sites in both regions. Such a review is also done for O isotope measurements made on humans from both Kuyavia and Nida Basin, and from a wider geographic context, especially for sites where both O and Sr isotope analyses were performed. Such a spatial extension of the review was necessary for two reasons. First, the number of studies based on O isotope analyses is low, and because measured O isotope values for early historic and later sites might be impacted by water processing, such as brewing or fermentation, commonly practised since Middle Ages. Second, the spatial variability of O isotopes in precipitation is relatively low and significant differences might only be visible between more distant regions. For that reason, also a map of estimated annual *δ*^18^O isotope ratios in precipitation is used to evaluate the values measured for humans in this and other studies.

### Sr isotope background of Kuyavia

**Fig 4:**
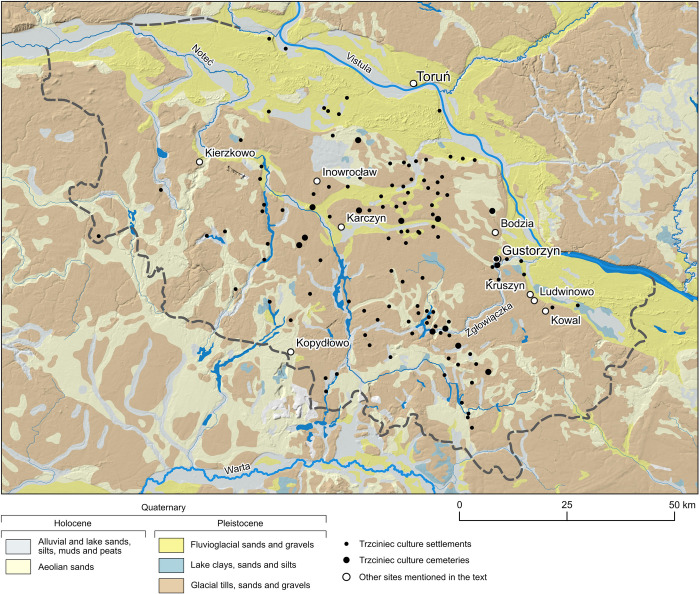
Geological map of Kuyavia with location of the Gustorzyn site, other cemeteries and settlements of the Trzciniec cultural circle societies, and other sites mentioned in this study. The map was produced with the QGIS 3.26.0 software, based on data from the Geological Map of Poland [129], and on data downloaded from https://www.hydrosheds.org/products/hydrorivers (License Agreement) and https://geoportal.gov.pl. Borders of Kuyavia and at the same time the area systematically surveyed by archaeologists is marked by grey dashed line.

Located in north-central Poland, Kuyavia is a relatively small region (ca. 8,000 km^2^) with vague borders defined on historical and ethnographic bases. Kuyavia consists of five main sub-regions (the Kuyavia Plain, Kuyavia and Żnin Lakelands, Toruń and Płock Basins) and is covered by Pleistocene glacial sediments of the Vistulian ice sheet and alluvial deposits of Holocene age [125, 126] (Fig 4). The glacial sediments are highly variable in lithology from clays to coarse-grained deposits and tills. They all consist of allochthonous clastic material, derived entirely from Scandinavia. This material is a product of glacial erosion and weathering of Precambrian igneous and metamorphic rocks of the Scandinavian Shield and, to a lesser extent, Palaeozoic and Mesozoic sedimentary rocks. The dominance of material from acidic igneous and metamorphic rocks is the reason for the high (radiogenic) ^87^Sr/^86^Sr ratios of the Pleistocene bedrock of the Kuyavia region, analogous to the ratios (average 0.730) observed in Precambrian rocks in the Fennoscandia region [127, 128]. Glacial sediments are thus the most radiogenic element of the local environment, with ^87^Sr/^86^Sr values everywhere above 0.72 (for review see [68]). The Kuyavia Plain, where Gustorzyn is located, consists of tills, weathered tills, and glacial sands and gravels.

The Kuyavia Plain is an undulating ground moraine, with melt-out depressions, ice-marginal valleys and subglacial channels. The latter are filled with lakes, of which Gopło is the largest. The biggest river is Noteć, followed by Parchania and Bachorza and their tributaries: Tążyna and Zgłowiączka (Fig 4), respectively. In contrast to the geological substrate, the riverine water of the Noteć and its tributaries exhibits moderately radiogenic ^87^Sr/^86^Sr values, lowered by inputs of chemical fertilizers in agriculture and mine waters from an open-pit lignite mine [73]. In the southern part of the region, where the Noteć River in its upper course is less affected by anthropogenic activities, the ^87^Sr/^86^Sr ratio has a maximum value of 0.7127. Groundwater from two natural outflows in the Żnin Lakeland (west of the Kuyavia Plain) yielded values of 0.7116 and 0.7111 [130]. It can be assumed that in the historical times the Sr isotope composition of groundwater could have been at this level or even was slightly more radiogenic.

A characteristic feature of the Kuyavian geology is the presence of Permian salt diapirs that pierced the Mesozoic strata and thus in some places occur directly under the Quaternary cover [131]. They constitute sources of ascending brines that influence locally the composition of groundwaters. This is because the brines are characterized by an unradiogenic composition with ^87^Sr/^86^Sr values below 0.7091 [73]. However, the main source of unradiogenic Sr in Kuyavia is atmospheric water (rain and snow) which has ^87^Sr/^86^Sr value of ~0.7092 identical to that of seawater [132, 133].

### Sr isotope data for archaeological humans and animals from Kuyavia

A relatively large pool of comparative data is available for the Kuyavia region in terms of variability of ^87^Sr/^86^Sr ratios in skeletal remains of prehistoric and early historic humans and animals. For three humans from a Late Neolithic population from Kierzkowo in the Żnin Lakeland the range of ^87^Sr/^86^Sr values measured in 11 teeth ranged from 0.7117 to 0.7140 but the range for local humans probably did not exceed 0.7135 [130]. In the case of a Roman period population from the cemetery in Karczyn-Witowy (western Kuyavia Plain), possible non-local individuals (two out of 15) showed ^87^Sr/^86^Sr values of 0.7092 and 0.7131, while others exhibited values between 0.7104 and 0.7123 [134]. The next two sites are located in the eastern part of the Plain, close to Gustorzyn. For a single Final Neolithic burial in Kruszyn two enamel samples yielded values of 0.7110 and 0.7123 [41]. Much lower values were reported for an early medieval population from the cemetery in Bodzia. Most individuals (11 out of 12) yielded values from 0.7090 to 0.7118 interpreted as non-local and only the male with the ^87^Sr/^86^Sr signature of 0.7129 was considered to be local [37, 135].

Enamel samples of fauna from sites in the Kuyavia Plain (Karczyn, Kruszyn, Ludwinowo; *n* = 3) exhibit values in the range 0.7124–0.7126 [134], at a number of sites from eastern part of the Plain (Bodzia, Ludwinowo, Kruszyn) these were 0.7121–0.7131 [37], in its southern periphery, close to Kuyavia Lakeland, they were between 0.7117 and 0.7130 (*n* = 9) [136]. In the Żnin Lakeland enamel samples of pig, goat and fox (*n* = 3) ranged from 0.71296 to 0.71339, while a series of sequential samples along a single cattle tooth (*n* = 14) gave unradiogenic ratios, from 0.7108 to 0.7113, interpreted as evidence of non-local origins of that specimen [130].

In the light of discussed data, the majority of people buried in Gustorzyn (with ^87^Sr/^86^Sr signatures from 0.7112 to 0.7125) most probably lived within this part of Kuyavia. The listed data also illustrates that ^87^Sr/^86^Sr ratios measured for enamel of archaeofauna from a number of sites in Kuyavia were clearly higher than those measured for humans.

### Sr isotope background of the Nida Basin

**Fig 5:**
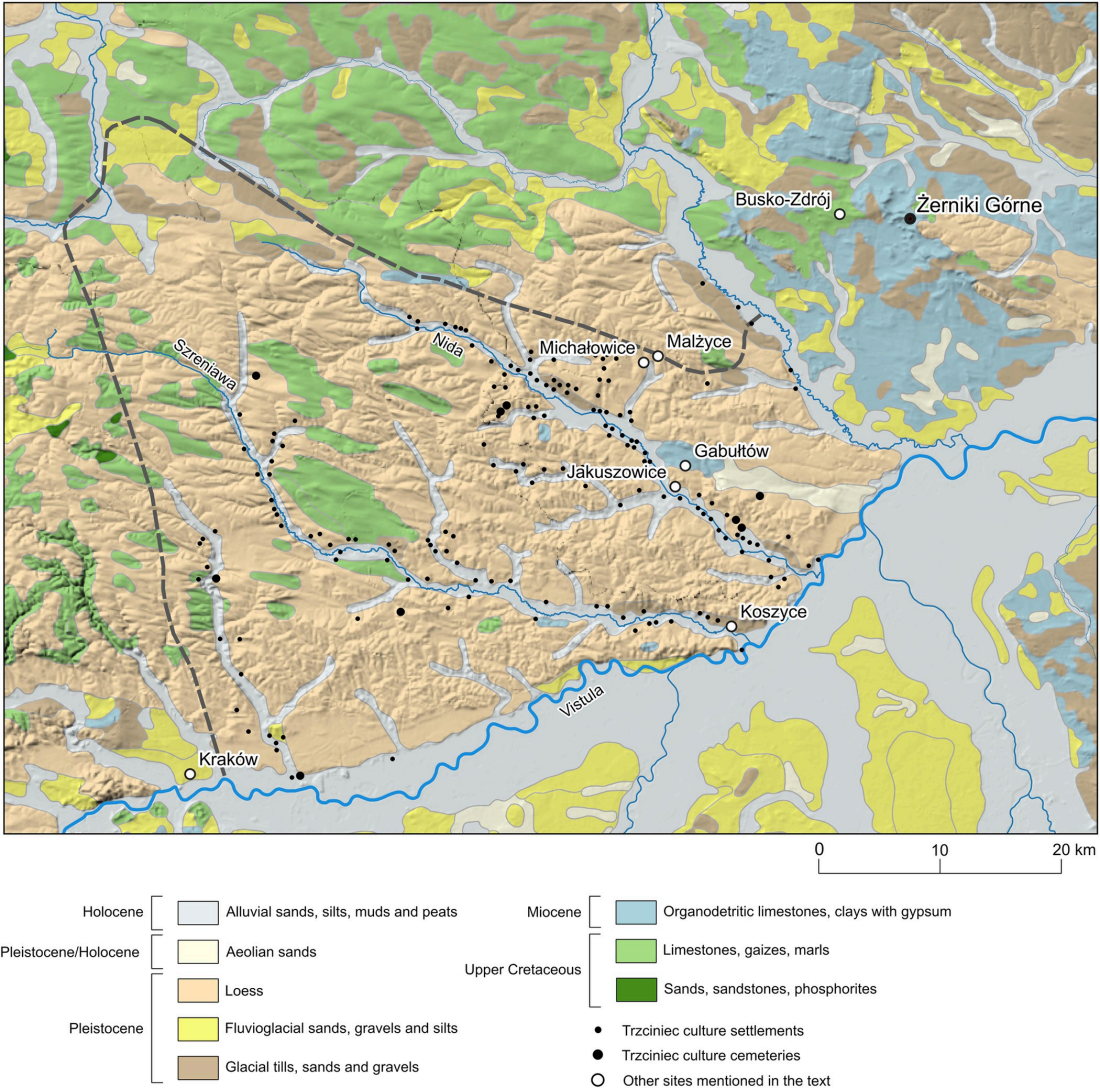
Geological map of southern Nida Basin with location of Żerniki Górne, other cemeteries and settlements of the Trzciniec cultural circle societies, and other sites mentioned in this study. The map was produced with the QGIS 3.26.0 software, based on data from the Geological Map of Poland [129], and on data downloaded from https://www.hydrosheds.org/products/hydrorivers (License Agreement) and https://geoportal.gov.pl/. Note that area systematically surveyed by archaeologists is marked by grey dashed line and it does not include the surroundings of Żerniki Górne.

The cemetery in Żerniki Górne is located in the central part of the Nida Basin which constitutes the marginal part of the Carpathian Foredeep Basin, developed during Miocene times due to thrust loading of the European foreland lithospheric plate by the Carpathian orogeny [137]. The sedimentary infill of the Nida Basin is represented primarily by thick, Miocene age argillaceous deposits (clays) containing layers of evaporates (gypsum). These rocks are widely exposed in the area around the town of Busko Zdrój (Fig 5). They overlay here a Mesozoic substrate composed of the Upper Cretaceous carbonates and marls. Northeast of Żerniki Górne, the Miocene rocks are covered by the Pleistocene deposits. Predominant among these are glacial tills and fluvioglacial sands which are Middle Pleistocene in age. Southeast of the site, the Pleistocene sequence terminates with a continuous cover of the Vistulian loess deposits [71, 138].

The Sr isotope composition of bedrock in the Żerniki Górne area is relatively well-known. ^87^Sr/^86^Sr values of 0.7088 to 0.7091 were reported for the Miocene gypsum deposits [139]. Recently, [71, 138] provided Sr isotope data for Miocene clays (values from 0.7144 to 0.7152) and Vistulian loess deposits (values from 0.7200 to 0.7240) exposed in the Nida Basin. The Sr isotope composition of the Upper Cretaceous substrate has not been measured so far. However, as these are marine carbonate rocks unradiogenic Sr signatures between 0.7074 and 0.7077 are to be expected [132]. Hence, around the investigated site at Żerniki Górne, an extremely wide variation in Sr isotope composition appears to characterize the local environment, from strongly unradiogenic to highly radiogenic signatures. In contrast to the wide variation in Sr isotope signatures of rocks, the riverine waters exhibit everywhere an unradiogenic composition. Signatures of the Vistula, Szreniawa, and Nida rivers vary within a narrow range between 0.7084 and 0.7095 [68].

### Sr isotope data for archaeological humans and animals from the Nida Basin

For the Nida Basin Sr data for humans is available from three sites. The first one is a Late Neolithic mass grave from Koszyce [71, 138]. It contained the remains of 15 people. ^87^Sr/^86^Sr values measured for all individuals range from 0.70963 to 0.71201, values for three samples of pig tooth enamel are between 0.70948 and 0.71126. It was proposed that humans with low values (0.7105–0.7110) are local, and those with more radiogenic Sr isotope composition (0.7113–0.7120) were born outside Koszyce area.

The next two sites are dated to Final Eneolithic. ^87^Sr/^86^Sr values for three humans from Malżyce, located in a loess upland ranged from 0.70954 to 0.71028 [71]. 13 individuals from Gabułtów exhibit Sr isotope ratios ranging 0.70956 to 0.71039 [71]. This cemetery is located further south from Malżyce, in an area dominated by Miocene clays. Despite differences in geological settings both groups exhibit similar ^87^Sr/^86^Sr values. All humans from both sites were interpreted as being of local origin, which corresponds to the local character of pottery vessels and flint tools found in their graves [71].

The animal baseline samples come from Gabułtów itself (horse tooth, ^87^Sr/^86^Sr value of 0.71026), and to locations in a loess upland, Michałowice (a horse tooth with ^87^Sr/^86^Sr value of 0.71117) and Jakuszowice (various domesticates with ^87^Sr/^86^Sr values ranging from 0.70996 to 0.71233). Due to possible high mobility of horses their values are not especially useful for establishing local baseline values.

The very narrow range of human ^87^Sr/^86^Sr values in Żerniki Górne (0.7082–0.7091) perfectly matches the isotopic composition of the Miocene sediments that form the main element of the local geological substrate. There is no direct information on the Sr isotope composition of surface and/or groundwater waters at Żerniki Górne. However, they are anticipated to be unradiogenic in composition as the nearest large river, Nida, draining a substrate with a similar lithological composition 15 km to the west, displays ^87^Sr/^86^Sr values from 0.7084 to 0.7091 [68]. As the Quaternary cover of radiogenic loess deposits is only rudimentary preserved in the eastern part of the Nida Basin (Fig 5), it can be assumed that the water available to people in Żerniki Górne was even less radiogenic. All of this provides compelling evidence for the local origin of all the individuals studied.

### Oxygen isotope baselines and variability across the two study areas

Oxygen isotope in bioapatite is typically used for tracking mobility over longer distances than Sr isotopes and owing to the low number of samples for oxygen isotopes, baselines and variability across both study areas are discussed here.

For Kuyavia *δ*^18^O values measured in various tissues were reported for two Late Neolithic humans from Kruszyn (*δ*^18^OC-VPDB –4.75 ‰, = *δ*^18^O_P-VSMOW_ 26.01 ‰ = *δ*^18^O_DW_ –7.27 ‰ [41]) and Kowal (*δ*^18^O_P-VSMOW_ from 20.86 to 25.23 ‰ = δ^18^O_DW_ from –1.60 to 5.13 ‰ [40]), and above mentioned early medieval population from Bodzia [37]. However, the majority of individuals from the latter site were assessed as being non-local based on their ^87^Sr/^86^Sr signatures so their *δ*^18^O values do not necessarily mirror local *δ*^18^O precipitation values.

*δ*^18^O_C-VPDB_ values have also been measured in tooth enamel of sheep, goat and sheep/goat from the Middle-Neolithic settlement in Kopydłowo located in the south of Kuyavia Plain [136]. The measurements were made along the M2 and M3 tooth profiles, which allowed to trace changes in O isotope ratios in the environment in an annual cycle. The obtained values after conversion to *δ*^18^O_P-VSMOW_ are in a range from 10.22 to 17.54 ‰ = *δ*^18^O_DW_ from –17.98 to –6.70 ‰. For an Early Neolithic LBK settlement in Ludwinowo (eastern Kuyavia Plain) ten cattle M3 teeth were sequentially sampled. Complete data for these samples has not been published but reported *δ*^18^O_C-VPDB_ values are between –8.4 and –2.5 ‰ [140], = *δ*^18^O_P-VSMOW_ from 13.3 to 19.6 ‰ = *δ*^18^O_DW_ from –13.3 to –3.6 ‰. As expected, O isotope composition in animals is much different than in humans, and values measured for the former cannot serve as a baseline for the latter.

Comparative O isotope data from Nida Basin is limited to above mentioned Final Neolithic cemetery in Malżyce [42] (Table 3). In addition, oxygen isotopes have been measured for humans buried at an early medieval (11^th^ c. AD) cemetery in Kraków [38], i.e., outside the Nida Basin but close to its western border (Fig 5). Samples of femur were collected for 65 individuals, including 11 children older than 5 years (most likely no longer breastfeeding) and 54 adults (22 males and 32 females).

**Table 3:** Annual *δ*^18^O_DW-VSMOW_ values estimated using Online Isotopes in Precipitation Calculator (OIPC [115]) and *δ*^18^O_P-VSMOW_ ratios measured for skeletal tissues of humans from prehistoric and early historic sites in Poland and north-eastern Germany discussed in this study (reported as *δ*^18^O_P-VSMOW_ or calculated from *δ*^18^O_C-VPDB_ values). *δ*^18^O_DW_ values for humans calculated from measured *δ*^18^O_P-VSMOW_ and *δ*^18^O_C-VPDB_ values. In case of humans O isotope composition was measured in single samples of enamel, except the individual from Kowal, for whom enamel, dentin and bones samples were analysed. Location of Tollense after [145], simplified.

site	N	E	m a.s.l.	OIPC *δ*^18^O_DW_ (‰)	*n*	mean *δ*^18^O_DW_ (‰)	sd	Δ OIPC–bioap *δ*^18^O_DW_ (‰)	min *δ*^18^O_DW_ (‰)	max *δ*^18^O_DW_ (‰)	median *δ*^18^O_P_ (‰)	mean *δ*^18^O_P_ (‰)	sd	min *δ*^18^O_P_ (‰)	max *δ*^18^O_P_ (‰)	references
Żerniki Górne	50°27’58”	20°47’33”	310	–9.3	18	–9.0	0.5	–0.3	–10.2	–8.3	16.1	16.1	0.3	15.3	16.5	this study
Gustorzyn	52°38’57”	18°53’33”	78	–9.2	15	–7.0	1.1	–2.2	–9.2	–5.5	17.4	17.4	0.7	15.9	18.3	this study
Tollense	53°44’53”	13°18’20”	0–6	–8.4	52	–7.8	1.3	–0.6	–10.3	–4.6	16.7	16.8	0.8	15.6	18.9	[14]
Bodzia	52°42’16	18°53’	87	–9.2	12	–6.9	1.1	–2.3	–9.5	–5.7	17.5	17.4	0.7	15.7	18.2	[37]
Górzyca	52°29’14”	14°38’47”	16	–8.3	10	–7.3	1.3	–1.0	–8.6	–4.7	16.9	17.2	0.8	16.3	18.9	[39]
Drawsko	52°51’19”	16°03’05”	50	–8.6	58	–6.9	1.1	–1.7	–9.5	–4.6	17.3	17.4	0.7	15.7	18.9	[35]
Malżyce	50°22’47”	20°29’52”	305	–9.2	13	–2.1	2.0	–7.1	–4.8	0.6	21.0	20.5	1.3	18.8	22.3	[71]
Kowal	52°32’25”	19°03’21”	95	–9.3	5	2.3	–2.9	–11.6	–1.6	5.1	23.0	23.4	1.9	2.3	–2.9	[4]
Kraków	50°03”42”	19°56’14”	210	–8.9	65	–10.0	2.1	1.1	–14.9	–4.6	15.5	15.4	1.4	12.23	18.91	[38]

**Fig 6:**
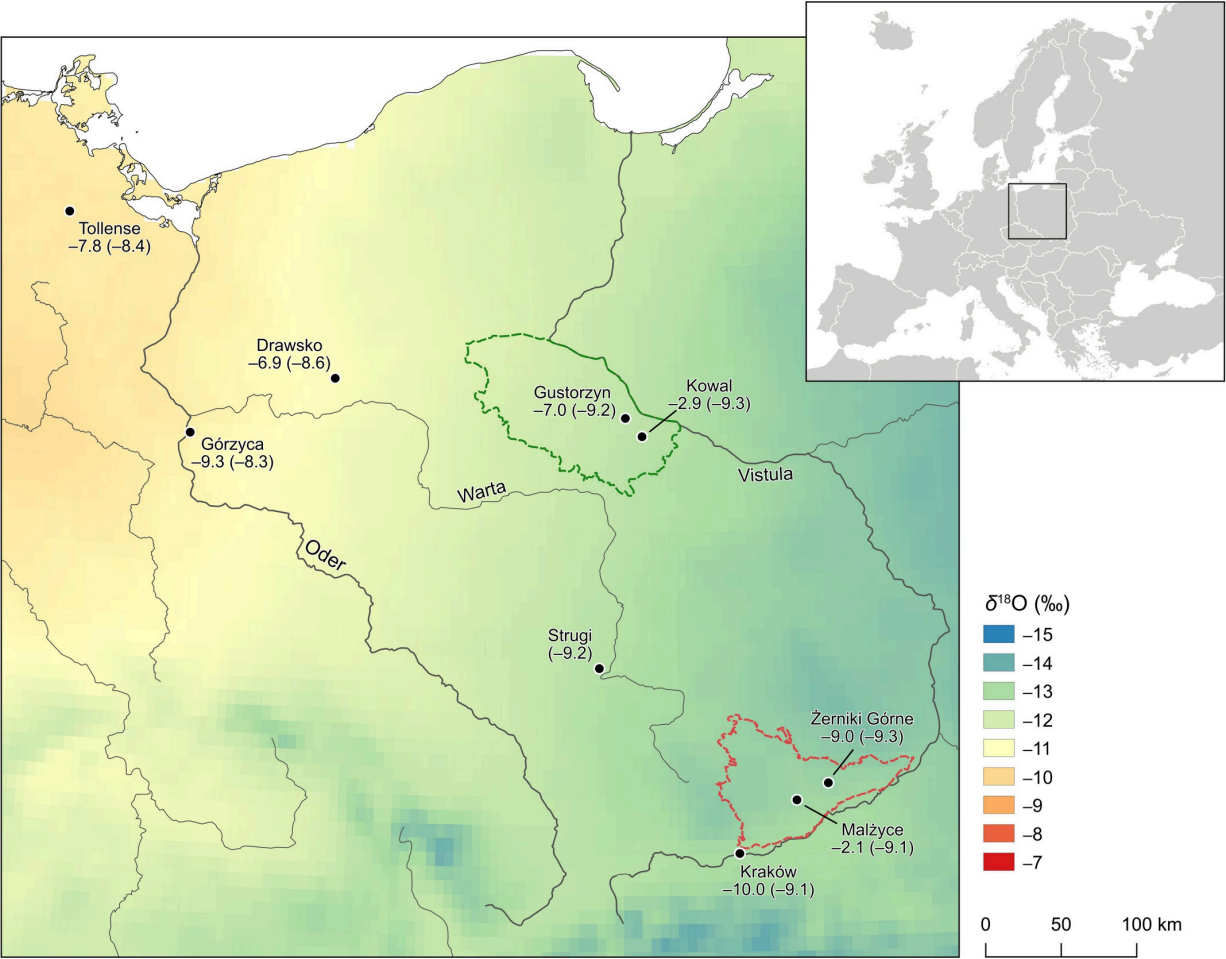
Map of estimated annual *δ*^18^O isotope ratios in precipitation in East-Central Europe. The map was produced with the QGIS 3.26.0 software, based on data from Online Isotopes in Precipitation Calculator (OIPC) database (v3.2, waterisotopes.org). Measured and/or estimated (in brackets) mean *δ*^18^O_DW_ values for humans from selected sites based on data from Table 3. Inset shows location of the study area in Europe.

To aid in the interpretation of the data and to help estimate the local baselines and variability a precipitation *δ*^18^O isoscape for the study regions (Fig 6) was generated using raster grid from OIPC v3.2 database [115], based on data from the Global Network of Isotopes in Precipitation and method of [141]. We accept that this database uses modern precipitation *δ*^18^O data which may not be strictly comparable to past *δ*^18^O_DW_ values but nevertheless represents a reasonable place to start estimating past *δ*^18^O_DW_. Fig 6 shows a strong Northwest-Southeast gradation in precipitation *δ*^18^O. Despite being nearly 300 km apart in latitude sites in east Kuyavia, near Gustorzyn, and in the Nida Basin have a similar and narrow range in precipitation *δ*^18^O from –9.3 to –9.1 ‰ (Table 3; Fig 6), in the North this trends to lower values westward towards Drawsko [35] and Górzyca [39].

**Fig 7:**
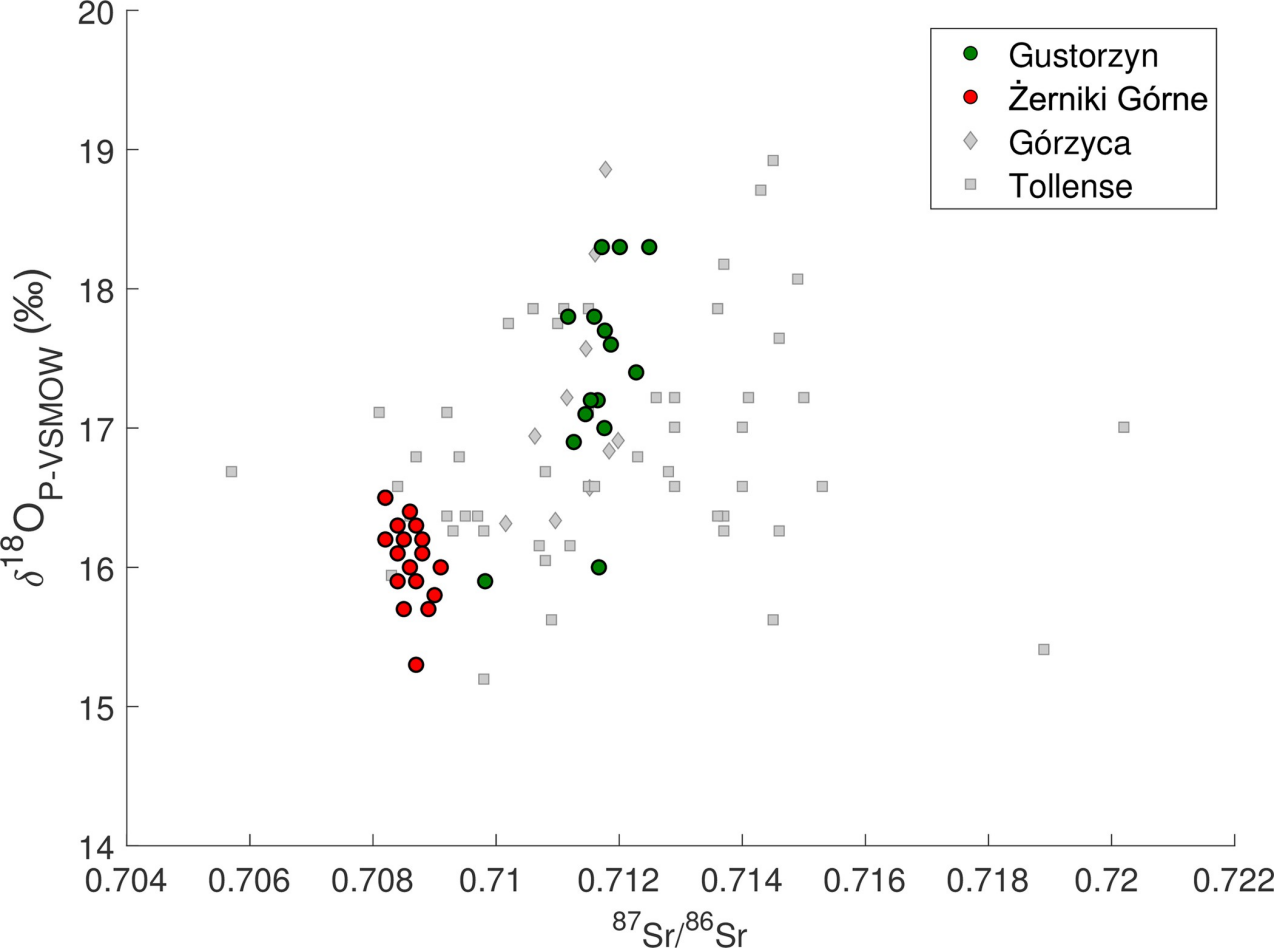
Strontium (^87^Sr/^86^Sr) and oxygen (*δ*^18^O_P-VSMOW_) isotope composition in human remains from Gustorzyn and Żerniki Górne (this study), and from Middle-Late Bronze Age sites in Górzyca [39] and Tollense [14].

Using estimated *δ*^18^O_DW_ values for the site from the model isoscape it is possible to calculate for each site Δ OIPC–bioap *δ*^18^O_DW_ value which is the difference between the model *δ*^18^O_DW_ and the measured mean *δ*^18^O_DW_ for humans, based on converting *δ*^18^O_P_ or *δ*^18^O_C_ to *δ*^18^O_DW_ values using Eqs (3) and (6) respectively. For most sites across the study areas the mean Δ OIPC–bioap *δ*^18^O_DW_ is <2.3 ‰ which is small in the context of the considerable error (>1 ‰) associated with the conversion of *δ*^18^O_P_ and *δ*^18^O_C_ values to *δ*^18^O_DW_. For Gustorzyn and Żerniki Górne Δ OIPC–bioap *δ*^18^O_DW_ value is –2.2 ‰ and –0.3 ‰ respectively, at the nearest Bronze Age site with O isotope data, Górzyca [39] (Fig 7) Δ OIPC–bioap *δ*^18^O_DW_ is also small at –1.0 ‰ (Table 3). The second Bronze Age population at Tollense, cannot serve as a benchmark here, as many of the studied humans were of non-local origins [14] (Fig 7). In the case of an early modern population from Drawsko [35] (Fig 6) significant processing of drinking water can be expected, leading to a gap between estimated and measured values. Still, this difference is low (–1.7 ‰) (Table 3). Comparative O isotope data in the Nida Basin is limited, however, for the medieval people from Kraków Δ OIPC–bioap *δ*^18^O_DW_ value is low (1.1 ‰).

Across both study regions, two oxygen isotope data sets standout as having very high Δ OIPC–bioap *δ*^18^O_DW_ values and are therefore in poor agreement with the modelled *δ*^18^O_DW_ values, these are the individual from Kowal in Kuyavia and the individuals from Malżyce in the Nida Basin.

For the individual from Kowal *δ*^18^O_P_ values have been measured on enamel, dentine and bone. Considering only the two enamel measurements on the first incisor and premolar, *δ*^18^O_P_ values are 25.23 and 22.21 ‰ respectively, giving a *δ*^18^O_DW_ value of –12.1 ‰ which is far outside the range for this part of East-Central Europe (Fig 6). This has already been noted by authors of the Kowal study [40]. Such a large discrepancy is difficult to be explained and the authors seem to be aware of that as they did not explicitly support the local or non-local origin of that individual [40]. Moreover, this single individual exhibits a greater range in oxygen isotope values (SD = 1.9) than all other populations in our study area.

For the site of Malżyce, the 13 individuals measured have mean *δ*^18^O_P_ values of 20.5 ‰, significantly high when compared to the nearby site of Żerniki Górne with a mean *δ*^18^O_P_ value of 16.1 ‰. Moreover, their Δ OIPC–bioap *δ*^18^O_DW_ of –7 ‰ is surprisingly low when compared to other sites, except Kowal (Table 3; Fig 7). What is also clear is the data from the Malżyce site exhibit a significantly greater range of *δ*^18^O_P_ values (SD = 1.3) (Table 3) compared to the contemporary site of Żerniki Górne (SD = 0.3) and all other sites discussed here (SD between 0.7 and 0.8), again except Kowal and Kraków (Table 3). Whilst it cannot be excluded that such a high variability of values for Malżyce reflects highly variable provenance of people buried there, similar cases are unknown to us. Even people recovered from Tollense battlefield exhibited lower variability in O isotope composition (SD = 0.8) [14] compared to Malżyce. This is important as their strontium, carbon (*δ*^13^C_C_) and lead (^204^Pb, ^206^Pb, ^207^Pb, ^208^Pb) isotope ratios clearly indicate they came from many regions, often very distant from the place of their death [14]. High *δ*^18^O_P_ variability (SD = 1.4) was also found among humans from Kraków [38].

For Malżyce and Kowal *δ*^18^O_P_ analyses were carried out for a mixture of enamel, dentin and bone samples, while for Kraków bone samples were analysed exclusively which is a rare case. For instance, a comprehensive meta-study of medieval sites from England and continental Europe [142] brings only one case when oxygen isotopes were measured in bones [143]. Here humans from an early medieval cemetery in Volders (Austria) can be characterised with a wide range of *δ*^18^O_C_ values between –16 and –5 ‰. After conversion to *δ*^18^O_P_ they range from 5.3 to 16.9 ‰ with a significantly high SD of 2.1. We are aware of two other studies based on bones. A medieval cemetery in Gródek at today’s Polish-Ukrainian border [36] again brings *δ*^18^O_P_ data of high variability with SD of 1.2 (Table 3). However, in a study of the early medieval cemetery in Povegliano Veronese in Norther Italy both enamel and bone samples of the same individuals were analysed, and bone samples had a significantly lower range of *δ*^18^O_P_ values than enamel [144]. Hence, it is currently inconclusive if *δ*^18^O values in bone can be more variable than in enamel. Moreover, we cannot explain why humans from Kowal and Malżyce exhibit much higher *δ*^18^O_P_ values than both: values expected from the OIPC model, and values measured for humans (both in carbonates and phosphates) from nearby archaeological sites. Hence, hereafter we will not refer to data for Kowal, Malżyce and Kraków (together with Gródek all coming from the same laboratory).

### Mobility in the Trzciniec cultural circle societies

The isotopic data from Gustorzyn shows that the majority of studied individuals are consistent with a local origin. The suspected non-local individual is a female aged 20–30 (individual 6 from grave 5). She exhibits outlying ^87^Sr/^86^Sr values (0.7098) and also the lowest *δ*^18^O _P-VSMOW_ (15.9 ‰ = *δ*^18^O_DW_ –9.2 ‰) value for the site. Her ^87^Sr/^86^Sr value is lower than the majority of prehistoric and early historic humans from Kuyavia who have been assessed as local in origin. A lower value (0.7092) was noticed only for 16–18 old individual of unknown sex from the Roman Period cemetery in Karczyn-Witowy [134]. Her ^87^Sr/^86^Sr value is consistent with an origin on relatively unradiogenic marine bedrock and with *δ*^18^O_DW_ values in between those which are characteristic for, for example, Kuyavia and the Nida Basin. One such area is the borderland of Greater Poland and Polish Jura, in the Upper Warta River basin, located up to 150 km S-W from Gustorzyn and known for clusters of TCC sites [45, 146]. *δ*^18^O_DW_ values for the southern-most cemetery of that cluster, in Strugi, can be estimated as –9.2 ‰ (Fig 6), which well fits the value received for the discussed woman. Here, the bedrock is Pleistocene sediments, locally eroded exposing Middle Jurassic marine rocks. Hence, one can expect relatively unradiogenic ^87^Sr/^86^Sr, which would be consistent with the hypothesis that this was a region where the woman spent her childhood. Alternatively, her Sr and O isotope ratios could be a result of migration from an area located further south, for instance in southern Poland, with lower Sr, although with similar O isotope values. If her journey took place during the period of enamel mineralization as an infant, her isotopic signatures would be a result of mixing the initial Sr and O values with higher values on the way North, although this is speculation. The non-local origin of the women from Gustorzyn may suggest the presence of female exogamy in the studied population. This was also noted by anthropometric analyses of cranial proportions [48, 56], although more isotopic information is needed to determine if this is an exceptional case or a more widespread practice.

The second individual from Gustorzyn with relatively low O isotope ratio is a child (individual 5) with *δ*^18^O_P_ values of 16.0 ‰ and an ^87^Sr/^86^Sr of 0.711670 which is consistent with a local origin. The interpretation of this individual is complex. There is a distinct possibility that this individual was breastfed, and this should lead to elevated *δ*^18^O_P_ values compared to the *δ*^18^O_P_ values expected for local adults i.e., the O isotope composition in local drinking water would have to be even lower than the equivalent of *δ*^18^O_P_ 16.0 ‰ = *δ*^18^O_DW_ –9.1 ‰. This would indicate that this child was of non-local origins and came from a region of relatively low *δ*^18^O_DW_ values, but with similar geology to Kuyavia Plain. Such a scenario is, however, rather unlikely. Britton and collaborators [112] found that infants from a medieval cemetery at Wharram Percy, England, exhibited elevated *δ*^18^O_P_ values in their deciduous teeth. However, already after the age of three, *δ*^18^O_P_ values measured in bones were >1 ‰ lower, similar to adult mean values or even lower than the equivalent local *δ*^18^O_DW_ values at the age 4–5 [112].

It is a challenge to assess what is the rate of mobility of the “local” people from Gustorzyn. Their ^87^Sr/^86^Sr values indicate that they had to have spent their childhood in an area covered by glacial sediments, which cover a large part not only of Kuyavia but also most of today’s northern Poland [129]. However, we observed that humans and animals from the flat moraine of the Kuyavia Plain exhibit distinctly less radiogenic Sr isotope ratios than majority humans from neighbouring regions, where ^87^Sr/^86^Sr values might exceed 0.7135 [37, 147]. Hence, we can suppose that people buried at Gustorzyn most probably limited their activity to eastern Kuyavia. In such a case they could exploit the area immediately surrounding the cemetery, and settle along Zgłowiączka, the nearest larger source of drinking water. Hence, the isotopic data is in agreement with the *site-catchment* model, assuming human activity within a radius of 2 km from the central settlement and nearby cemetery.

*δ*^18^O_P_ signatures (16–18.3 ‰) of people from Gustorzyn are not very helpful in assessing their short-range mobility, as similar values (15.7–18.9 ‰) were received for a cemetery in Drawsko [35], located as far as 190 km to the West (Fig 6). Next cemetery with O data, Górzyca, is located 280 km West from Gustorzyn, and also has similar values from 16.3 to 18.9 ‰ [39] (Table 3). In other words, the variability of O isotopes in this part of Europe is insufficient to trace journeys on the East-West axis shorter than at least 300 km. Travels along North-South axis are easier to detect, as *δ*^18^O_DW_ values are more variable due to larger changes in altitude and distance from the sea. Moreover, the equipment of both graves at Gustorzyn does not include any non-local objects which could indicate that one of the buried humans came from regions outside the TCC-world. The most distinctive, cultural tradition contemporary with the “Trzciniec” was the Tumulus culture, covering regions located further West and South-West, the nearest being Greater Poland and Silesia [146, 148]. The stylistics of bronze items from Gustorzyn resemble the Tumulus culture style but such southern traits are common in Kuyavia and point towards intensive interactions with the neighborhood rather than migration(s) [148]. They were preceded by trans-Carpathian influences from the Otomani-Füzesabony circle, though they were less intense here than in the Nida Basin [149].

In contrast to Gustorzyn, it is highly probable that all studied individuals from Żerniki Górne are of local origin and spent their infancy close to the place where they were ultimately buried. Some of the communal graves at this site contain equipment that might evidence influences of other cultural groups [51]. This is a broader regularity, also visible at other sites in the uplands of southern Poland and associated with intense contacts between local elites and highly developed communities of the Otomani-Füzesabony culture from the Carpathian Basin and its northern edges [29, 149–153]. The style of pottery vessels (types of containers, specific ornaments) and bronze items were clearly inspired by “Southern” patterns. Although the exogamy between the TCC populations from Nida Basin and the Otomani-Füzesabony culture communities settled in the Subcarpathian zone cannot be ruled out, the most reliable interpretation of similarities in material culture is far-reaching exchange of amber and metal [21, 22, 29, 153].

The cemetery in Żerniki Górne is located at the edge of two areas with distinct lithologies, with Pleistocene loess prevailing in the East, and Miocene clays and evaporates covering lower parts of the landscape within a radius of at least 5 km around the site (Fig 5). As all humans exhibit unradiogenic ^87^Sr/^86^Sr ratios, which are typical for the Miocene geology, these people most probably occupied these lower areas. It can be hypothesised that they settled at Nida River, flowing 15 km South-east from the site, and draining non-radiogenic Cretaceous bedrock. The latter would be the most plausible source of water with unradiogenic Sr. This can also be seen the other way round: Sr isotope composition in human tooth enamel allows to predict the location of an area exploited by these humans. This stays in agreement with archaeological observation of settlements being located along rivers, and cemeteries occupying higher parts of the landscape (Fig 5).

The range of ^87^Sr/^86^Sr values for humans from Żerniki Górne is extremely narrow when compared to other human populations, both in Nida Basin and Kuyavia. This might indicate that the area they exploited for farming and husbandry was geologically homogeneous. However, considering the relatively high variability of lithology in radius of ~10 km around the site, their mobility was most probably limited to very specific and rather small parts of the landscape. As in case of Gustorzyn, O isotope data is not helpful in testing short-distance mobility of the studied humans. Nevertheless, it allows to reject the possibility of long-distance travels from regions with similar geological settings as Żerniki Górne which would lead to ^87^Sr/^86^Sr ratios indistinguishable from the local signal.

## Conclusions

From among 34 investigated individuals, only the woman from Gustorzyn in Kuyavia can be considered non-local. It seems most likely that she came from an area further to the South, possibly from the region of TCC settlements concentrated in the upper Warta River. Both the Sr and O signatures indicate her non-locality. The obtained results confirm the earlier hypotheses put forward by archaeologists, namely that mobility in the “Trzciniec” populations was small and limited to individual people or small family groups. Importantly, despite the features of material culture indicating the trans-Carpathian contacts of the TCC societies, both in Kuyavia and Nida Basin, only a single case migration between these two regions was found.

The non-local origins of a woman may also indicate the existence of female exogamy in the TCC societies. However, it should be remembered that migration was found for only one individual and the number of all studied individuals is relatively small. Therefore, it is not possible to draw too far-reaching conclusions on this basis and formulate broader generalizations. On the other hand, it cannot be ruled out that exogamy was more common in the studied populations, but it occurred between populations living in closely located areas and with similar geological structure and climatic conditions. As a result, the differences in residence may not be visible in the measured ratios of Sr and O isotopes.

The currently available isotopic data indicate that the area of the Nida Basin under consideration can be divided into two parts with different geological settings, which translates into different ranges of bioavailable ^87^Sr/^86^Sr values. The TCC populations inhabiting its western part, covered mainly with loess, show Sr values in the range from 0.7095 to 0.715. The eastern part, mostly composed of marine sedimentary rocks and clay, is much less well known both archaeologically and in terms of the variability of bioavailable Sr isotope ratios. The pilot data obtained for people from the cemetery in Żerniki Górne suggest that the local populations in this area would show values ranging from 0.708 to 0.709. Thus, both within Nida Basin and to a lesser degree in Kuyavia, it is possible to use Sr isotopes to detect people’s travels over relatively short distances if only they crossed borders between clearly marked isoscapes after the period of enamel mineralization.

The examination of a sufficiently large number of individuals from two locations allows us to consider the results of Sr isotope measurements as reliable. Both the distribution of values and the comparison with environmental data clearly indicate the limited mobility and local origins of the majority of individuals in the period between 16^th^ to 13^th^ c. BC. The O isotope results are in agreement with the Sr isotope results, indicating limited mobility for most individuals. Moreover, the mean values ​for both populations, defined as local, are consistent with the predicted modern O isotope precipitation values. This highlights the utility of this method for tracking migrations over longer distances but also the problems with using O isotopes in the absence of suitable variations in *δ*^18^O_DW_ values.

## Supporting information

S1 Text Description of graves from the cemetery in Gustorzyn site 1 and Żerniki Górne site 1 included in this study. (DOCX)
