# Supplementary material for: Assessing the mobility of Bronze Age societies in East-Central Europe. A strontium and oxygen isotope perspective on two archaeological sites
Source: PLoS One. 2023 Mar 17;18(3):e0282472. doi: 10.1371/journal.pone.0282472 (PMC10022790; doi:10.1371/journal.pone.0282472)
Supplement: S1 Text — (DOCX) [file pone.0282472.s001.docx]

**S1. Description of graves from the cemetery in Gustorzyn site 1 and Żerniki Górne site 1 included in this study.**

**Gustorzyn site 1, grave no. 4**

A grave partially destroyed by a gravel pit, oriented along the NW-SE axis. Preserved length of the burial pit 1.0 m, with a width of approx. 0.80 m. The south-eastern wall touched the edge grave no. 5. It contained partially mixed up remains of at least 10 individuals: three individuals in the age of *Infans*, two individuals in the age of *Iuvenis*, three adults males, and two adults of unspecified sex (Szczepanek 2001, p. 293, Szczepanek 2002).

**Gustorzyn site 1, grave no. 5**

The grave was completely preserved. Burial pit had a rectangular outline with rounded corners, with the size of approx. 2.8 x 0.70 m, oriented along the N-S axis, and surrounded and partially covered with erratic boulders with a diameter from approx. 0.25 to 0.8 m. Human remains were in a partially preserved anatomical order, disturbed by post-depositional processes. At least 22 individuals were buried in the grave: seven children in the age of *Infans*, six females, seven males, two adults of undetermined sex (Szczepanek 2001, p. 293). Nine individuals were buried in the northern part of the grave, two in the middle and seven in the southern (ibid.). The dead were placed anti-modally, in the southern part of the grave more males were buried (four males, two females and one child), in the north – more females (one male, four females, two children, to adults of undetermined sex) (Szczepanek 2013, p. 76). The remains of women and children rested close to each other, in the centre of the grave there were remains of a male and a child (ibid.). In the middle part of the grave a bronze bracelet was found, in the southern part there was a bone pin and two ceramic vessels (Grygiel 1987, pp. 81-82, Figs. 8 and 9; Dąbrowski 2004, pp. 39, 48-50, 55).

**Żerniki Górne site 1, grave no. 10**

The burial pit was oriented along the W-E axis, readable only in the western part, with an outline close to rectangular, total length approx. 4.0 m, width approx. 0.75 m. It contained poorly preserved remains of 10 people. In the eastern part of the grave, five skulls were unveiled, in the western part one complete and fragments of the next ones were found. Bones of postcranial skeletons lay squeezed together and on each other. The dead were buried with strongly drawn up legs. In the eastern part of the grave a single amber bead was found (Kempisty 1978, pp. 168-169).

**Żerniki Górne site 1, grave no. 12**

The burial pit had a regular shape, with W-E orientation, approx. 2.5 m long and approx. 0.8 m wide. A wooden coffin with a rectangular outline and approx. 0.70 m wide was found inside. 3 limestone Three with a dimeter of approx. 0.20 m were placed between the coffin and the walls of the pit. At least eight individuals were buried in the grave. Four human skulls were found in the eastern part and four in the western part. Bones of postcranial skeletons lay squeezed together and on each other. In the eastern part of the grave, inside the coffin, a small pottery vessel and an amber bead were unearthed. In the southern part of the grave a bronze pin was found (Kempisty 1978, pp. 169-171).

**Żerniki Górne site 1, grave no. 62**

Burial chamber with NW-SE orientation, with an almost rectangular outline, 3.8 m long and an average width of 1.2 m. Nine people were buried inside. In the south-eastern part of the grave there were two skeletons placed on the left side, along the axis of the pit, with heads on the SE – the northern one with strongly drawn up legs, the southern one with the legs slightly bent. To the west of the first of them there was a skeleton placed on the right side, along the axis of the pit, but with the head to NW. To the south of him was a skull of an unidentifiable individual. In the widened north-western part of the grave, a badly preserved skeleton was unearthed, placed in a crouched position on the left side, with strongly drawn up legs, along the axis of the pit and with head to NW. In the north-western part of the grave there was a skeleton placed on the left side, along the axis of the pit, with head to NW, with slightly drawn up legs, and three skulls impossible to assign to specific postcranial skeletons. Three pottery vessels were found above the skeletons (Kempisty 1978, pp. 172-175).

**Żerniki Górne site 1, grave no. 69**

The burial pit had an almost rectangular outline, oriented along the NW-SE axis, approx. 4.7 m long and on average 1.6 m wide. Human remains lay in a cluster separated by empty space from the walls of the pit, in its north part there were four stones. 26 whole or fragmented human skulls were discovered in the grave and on this basis the minimum number of individuals buried in the grave was determined. In the northern part of the grave, 15 skulls and a cluster of bones of postcranial skeletons were unveiled. In the middle part of the grave a skeleton was found in a position on the left side, along the axis of the pit, with the head to NW, with slightly drawn up legs. In the southern part of the grave there were three skulls and bones of postcranial skeletons. Whole fragments of vessels were found throughout the burial pit. In the central part of the pit, an amber bead was found, and in the southern part, a fragment of a bone pin (Kempisty 1978, pp. 174-186).

**Żerniki Górne site 1, grave no. 99**

The burial pit with had an almost rectangular outline, NW-SE orientation, it was approx. 3.6 m long and on average 0.75 m wide. 21 people were buried inside. In the northern part of the grave seven skulls were discovered, the postcranial skeletons associated with them lay along the longer axis of the grave, with heads to NW, with strongly drawn up legs. In the south-eastern part of the grave, seven or eight skeletons in the same position were found, with heads to SE. Ceramic vessels as well as ornaments made of bronze, amber, glass, bone and other materials were found between the human bones (Kempisty 1978, pp. 206-215).

**References**

Dąbrowski, J. 2004. Ältere Bronzezeit in Polen. Starsza epoka brązu w Polsce. Warszawa.

Grygiel, R. 1987. Z badań nad kulturą trzciniecką w rejonie Brześcia Kujawskiego, In: P. Polewska, J. Rydzewski (eds.), Kultura trzciniecka w Polsce, Kraków, 73–89.

Kempisty, A. 1978. Schyłek neolitu i początek epoki brązu na Wyżynie Małopolskiej w świetle badań nad kopcami. Wydawnictwa Uniwersytetu Warszawskiego, Warszawa.

Szczepanek, A. 2001. Pochówki zbiorowe kultury trzcinieckiej z Bocheńca, stan. 2, woj. świętokrzyskie i Gustorzyna, stan. 1, woj. kujawsko-pomorskie – analiza antropologiczna. Sprawozdania Archeologiczne 53, 289–312.

Szczepanek, A. 2002. Analiza antropologiczna szkieletów ludności kultury trzcinieckiej z grobów zbiorowych z Gustorzyna, st. 1, woj. kujawsko-pomorskie, Prace i Materiały Muzeum Archeologicznego i Etnograficznego w Łodzi, Seria Archeologiczna 42, 53–76.

Szczepanek, A. 2013. Archeotanatologia pochówków zbiorowych od pradziejów po czasy współczesne, Rzeszów.
